# The importance of the neuro‐immuno‐cutaneous system on human skin equivalent design

**DOI:** 10.1111/cpr.12677

**Published:** 2019-08-23

**Authors:** Sarah E. Vidal Yucha, Kasey A. Tamamoto, David L. Kaplan

**Affiliations:** ^1^ Department of Biomedical Engineering Tufts University Medford Massachusetts; ^2^ Department of Chemistry Tufts University Medford Massachusetts

**Keywords:** human skin equivalent, in vitro model, neuro‐immuno‐cutaneous system, peripheral innervation, peripheral nerve, tissue engineering

## Abstract

The skin is a highly complex organ, responsible for sensation, protection against the environment (pollutants, foreign proteins, infection) and thereby linked to the immune and sensory systems in the neuro‐immuno‐cutaneous (NIC) system. Cutaneous innervation is a key part of the peripheral nervous system; therefore, the skin should be considered a sensory organ and an important part of the central nervous system, an ‘active interface’ and the first connection of the body to the outside world. Peripheral nerves are a complex class of neurons within these systems, subsets of functions are conducted, including mechanoreception, nociception and thermoception. Epidermal and dermal cells produce signalling factors (such as cytokines or growth factors), neurites influence skin cells (such as via neuropeptides), and peripheral nerves have a role in both early and late stages of the inflammatory response. One way this is achieved, specifically in the cutaneous system, is through neuropeptide release and signalling, especially via substance P (SP), neuropeptide Y (NPY) and nerve growth factor (NGF). Cutaneous, neuronal and immune cells play a central role in many conditions, including psoriasis, atopic dermatitis, vitiligo, UV‐induced immunosuppression, herpes and lymphomas. Therefore, it is critical to understand the connections and interplay between the peripheral nervous system and the skin and immune systems, the NIC system. Relevant in vitro tissue models based on human skin equivalents can be used to gain insight and to address impact across research and clinical needs.

## Graphical abstract

The skin is a highly complex, sensory organ composed of three main layers: epidermis, dermis and hypodermis, and numerous cell types which interact to make up the neuro‐immuno‐cutaneous (NIC) system. The skin is a sensory organ, responding to various stimuli through orchestrated cellular and molecular signals. The NIC system involves cell‐cell signalling and mediation from neuropeptides/neurotransmitters, cytokines and growth factors. HSEs, human skin equivalent; NIC, neuro‐immuno‐cutaneous.

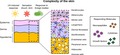

## 1 INTRODUCTION

**Figure 1:**
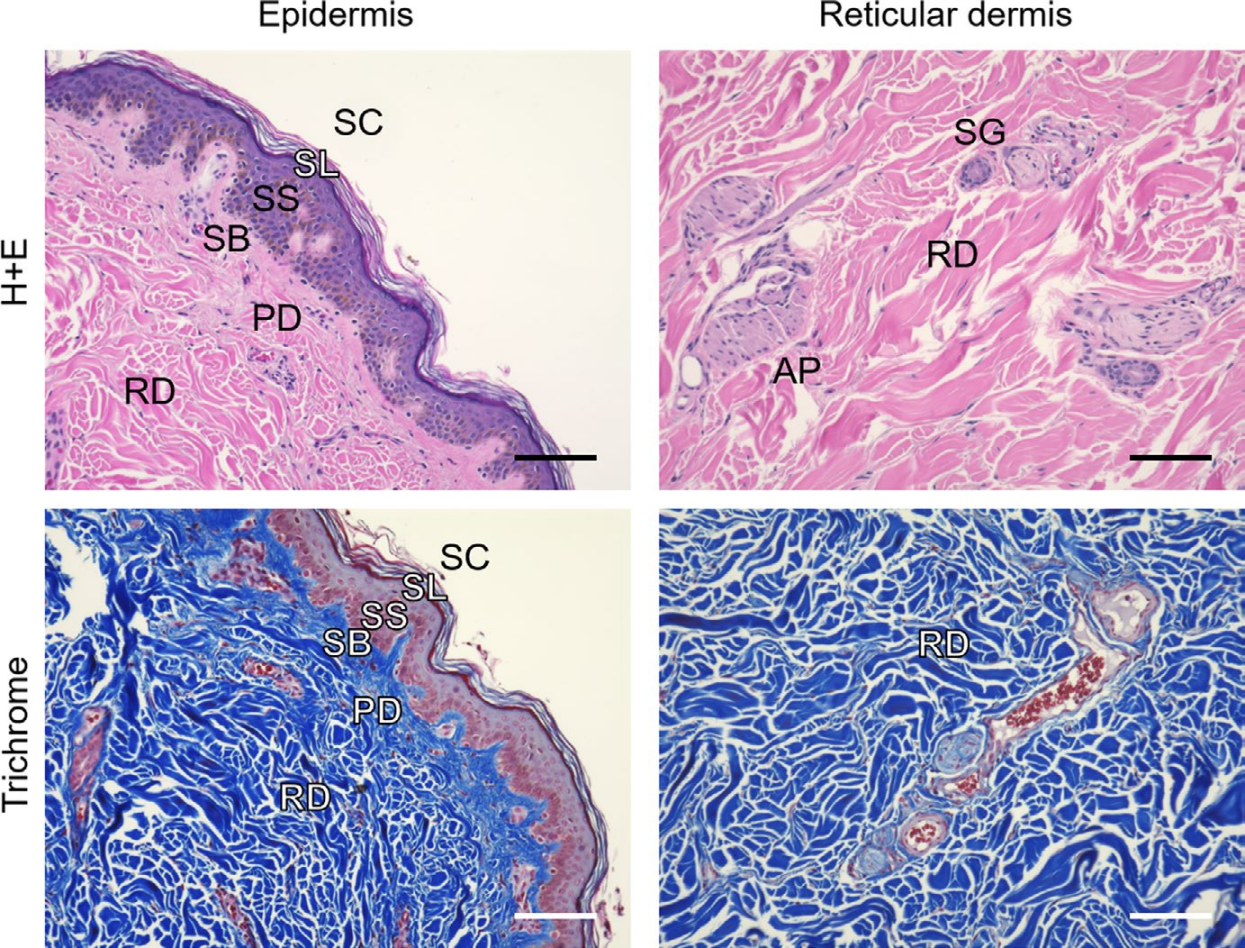
Complex nature of human skin. Skin biopsy obtained from abdominoplasty procedure (Tufts University IRB Protocol #0906007) at the Lahey Clinic (Burlington, MA, USA) demonstrates intricate tissue structure of the native skin. Abbreviations: SC, stratum corneum; SL, stratum lucidum; SS, stratum spinosum; SB, stratum basal; PD, papillary dermis; RD, reticular dermis; SG, sweat gland; AP, arrector pili muscle. Scales are 100 µm

The skin is the largest organ in the body and one of the most complex, as a multi‐layered, multiple cell type, multifunctional organ that serves as a key interface to the outside world.^1^ The skin is composed of three main layers: epidermis, dermis and hypodermis (subcutis). Each layer is composed of multiple cell types with unique and complementary functions to support homeostasis (Figure 1, Table 1). The epidermis contains keratinocytes, melanocytes, Langerhans cells and Merkel cells. The dermis contains dermal fibroblasts, mast cells, vascular smooth muscle cells, specialized muscle cells, endothelial and immune cells. The hypodermis is composed of adipocytes, nerves and fibroblasts. The complex functional components of these layers include sweat glands, hair follicles, blood vessels and peripheral nerve endings (Aβ, Aδ, and C nerve fibres).^1^, ^2^, ^3^, ^4^, ^5^, ^6^, ^7^, ^8^, ^9^, ^10^, ^11^

**Table 1:** Cell types and functional components of the skin with their location and known functions

Skin cell type or component	Location(s)	Function(s)
Keratinocyte	Epidermis	Epidermis is stratified into numerous layers with distinct function (basal, spinous, granular and uppermost stratum corneum)^70^; terminally differentiated keratinocytes of the outer epidermis play a role in immune modulation and also still communicate with stem cells, other skin cells and immune cells in the epidermal basal layer^71^ Keratinocytes of the stratum corneum produce lipids to serve, in part, as protective barrier layer but the microenvironment of the skin (ie lipid concentration, bacteria population/microbiota, moisturization) will be distinct with respect to location on the body^70^, ^72^ Keratinocytes deposit keratins, proteins responsible for numerous processes distinct through differentiation stages of the keratinocyte that also add mechanical strength to the skin^10^ Keratinocytes have neurotransmitter receptors, respond to neuropeptide activity in the skin, and re‐epithelialization can be stimulated via neuronal‐keratinocyte signalling^31^, ^43^, ^70^, ^73^
Melanocyte	Epidermis	Melanogenesis^74^ Secrete many signalling molecules including pro‐inflammatory cytokines, immune and neuromediators^74^, ^75^ Interact with keratinocytes which regulate many functions of melanocytes^74^ May be responsive to β‐amyloid with effect on cholinergic neurons, with implication in Alzheimer's disease^76^, ^77^
Langerhans immune cells	Epidermis	Important antigen‐presenting cell that diminishes with age and may be related to lack of cutaneous immune function in ageing patients^78^ Maintain immune homeostasis in skin can stimulate T‐cell population^79^
Merkel cells	Epidermis	Closely associated with dermal sensory neurons 80 and form Merkel‐neurite complexes with the Aβ nerve terminals^9^ Mechanosensation^10^ Suggestion that they may be sensory receptor cells themselves^81^ may be acted on by neurotransmitters 65
Dermal fibroblasts	Dermis	Type of fibroblast is becoming more important as they can have diverse function with respect to organ^82^ Secrete extracellular matrix and basement membrane proteins, mainly collagen I, III, IV, laminin, proteoglycans^10^ Papillary dermis located closest to epidermis and contains a higher concentration of dermal fibroblasts than other layers; reticular dermis is collagenous, fibrous support tissue^71^
Mast cells	Dermis	Neuropeptides can activate mast cells^39^ as can numerous other stress mediators^4^ Mast cells are often located close to sensory nerve and blood vessels in the skin, known as a first‐line defence immune cell that quickly and selectively respond to physiological stress^4^
Vascular smooth muscle cells	Dermis	Can produce pro‐inflammatory cytokine interleukin‐6 (IL‐6) in skin (along with keratinocytes, fibroblasts, endothelial cells, immune cells)^83^ Constrict blood vessels following injury^19^ Smooth muscle is closely associated with neurons and hair follicles^11^
Endothelial cells	Dermis	Angiogenesis^84^ Proliferation of endothelial cells (and fibroblasts) can be enhanced through adding structural/mechanical strength to dermal tissue^85^ Response to inflammatory or environmental events by secretion of cytokines including intercellular adhesion molecules (ICAM‐1)^86^ or interleukin‐8 (IL‐8)^39^ Endothelial cells are in close contact with neuronal cells in the skin and can respond to neuropeptide signalling 39
Immune cells (Macrophages, monocytes, eosinophils, basophils, neutrophils, T cells, dendritic cells, innate lymphoid cells)	Dermis	Mediate the innate immune system and inflammatory reactions^10^, ^87^, ^88^ The skin contains diverse dendritic immune cell population with functions in both healthy and diseased skin^49^, ^51^, ^52^ Allergic reaction^46^ Promote homeostasis or inflammation^50^ Immune system surveillance^10^
Sensory neurons	Epidermis, dermis, hypodermis	Afferents are further classified into Aβ, Aδ and C nerve fibres with defined roles in the skin related to their action‐potential propagation speed, a function of their degree of myelination^20^, ^27^, ^65^ Secrete neuropeptides, neurotrophins, neurohormones^8^, ^39^, ^40^ Sensation, touch, response to mechanical, chemical or thermal stimuli, ‘nociception’^9^, ^40^, ^65^ Pain, neurogenic‐inflammation^6^, ^28^, ^40^ Vascular regulation, vasodilation via sensory nerves, vasoconstriction via neuropeptide signalling^8^
Adipocytes	Hypodermis	Absorbs mechanical loads, insulates^10^ Mediates fibroblast recruitment during wound healing^89^ Energy source responsible for triglyceride production^90^ May function as endocrine organ through secretion of growth factors, hormones and cytokines to communicate with the rest of the NIC/NICE systems, associated with lipid metabolism and other metabolic processes^89^, ^90^, ^91^, ^92^ Adipocyte bi‐directional communication with neurons modulates metabolic (leptin production, lipolysis) and neuropeptide production^90^; sensory neurons may mediate adipose/cutaneous inflammation^93^

**Figure 2:**
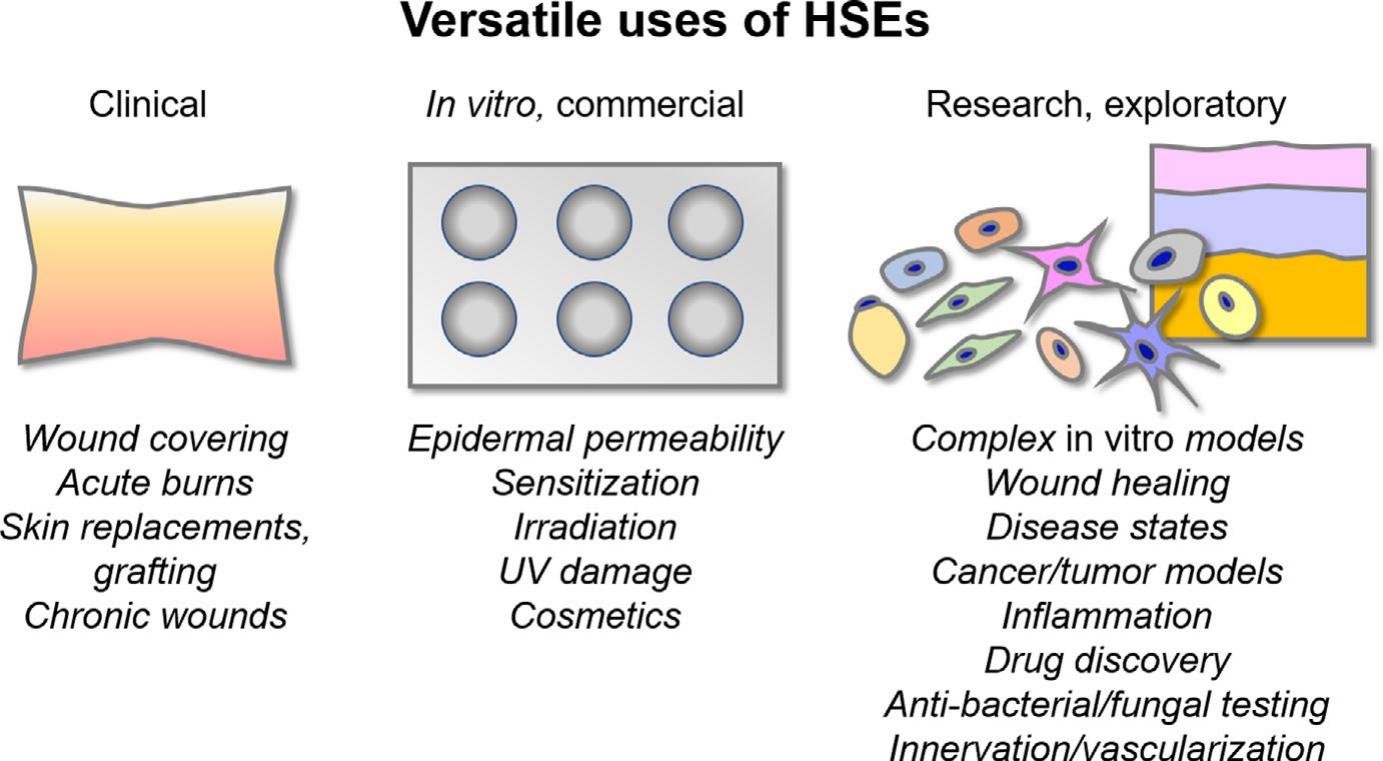
Versatile uses of HSEs. HSEs can be used for clinical, commercial or research applications, spanning different areas of interest. Complex in vitro HSE models can be used to address the interplay between NIC systems and enhance understanding of permeability and sensitization by including additional components (ie nerve, immune cells or additional skin cells typically not included in standard HSEs like melanocytes or Merkel cells)

Efforts in skin research are typically divided into three areas of importance: clinical models, commercial in vitro testing and exploratory research (Figure 2). Clinical research on skin focuses on the development of reliable human skin equivalents (HSEs) that can be used as dermal grafts, skin replacements, or wound coverings in acute cases, or for chronic cases that include diabetic ulcers or non‐healing wounds.^12^, ^13^ The general constraints for these models include that they must be biocompatible, integrate with the existing tissue beneath (ie subcutis/hypodermis), and interface with the surrounding tissue along the perimeter of the replacement, and they must be approved by the FDA, so material and regulatory concerns are met.

Clinical models have significantly advanced in the past decades along with the advent of tissue engineering, and currently, there are numerous options for clinicians to choose from, diverse in delivery format, in composition of cells or tissue, and in the choice of matrix material (Tables 2 and 3). The clinical research field is growing and expected to be valued at $24.3 billion USD by 2019.^3^ However, as the demand continues to grow, there are various problems which are still pertinent to the clinical field, including rejection, scarring, size constraints and lack of integration with functional components of the skin (ie sensation may not return due to lack of innervation, hair follicles or pores may not develop).^12^, ^13^

**Table 2:** Summary of some current commercially available human skin equivalents divided into epidermal, dermal or epidermal‐dermal composite replacements^2^, ^69^

Type	Selected method(s) delivery	Components
Epithelial cover	Integrated sheet (Epicell—Genzyme) Cell spray (CellSpray—Clinical Cell Culture)	Autologous keratinocytes
Dermal‐only replacements	Donor skin Synthetic material with fibroblasts (Dermagraft—Advanced Biohealing)	Screened donor dermis Donor fibroblasts
Epidermal/dermal replacements	Bovine collagen sheet containing cells (Apligraf—Organogenesis) and (Permaderm—Cambrex)	Allogenic (Apligraf) or autologous (Permaderm) keratinocytes and fibroblasts

**Table 3:** HSE models with classifications, applications, and challenges^2^, ^3^, ^5^, ^69^

HSE model classification	Sub‐classification	Example	Application	Current challenges
Commercial replacements	Epidermal Dermal Composite (Epidermal/Dermal)	Apligraf (Organogenesis) Dermagraft (Advanced Biohealing) Orcel (FortiCell Bioscience)	Burn patients, skin grafting/wound cover, chronic wounds	Scarring, rejection, integration of vasculature, nerve, hair follicles
Commercial in vitro systems	Epidermal—single layer Epidermal—full thickness Composite (Epidermal/Dermal)—full thickness Reconstructed human epidermis (RHE)	EpiSkin (SkinEthic) Epiderm (MatTek) Epiderm FT (MatTek) SkinEthic RHE (SkinEthic)	Drug development, wound healing, permeability, sensitization	Increasing test period window, increasing complexity for analysis
Research systems	2D monocellular 2D co‐culture 3D co‐culture 3D HSE (multiple cell type) In vivo (animal) In vitro human explants (cultured) In vivo human (skin grafts or biopsies) *In virtuo* model	2D monocellular^94^, ^95^ 2D co‐culture 32 3D co‐culture of keratinocytes and T cells^96^ 3D HSE^31^, ^97^ In vivo mice^98^, ^99^ In vitro human explants (cultured), skin grafts or biopsies^100^ In vivo human (skin grafts or biopsies)^101^, ^102^ Computational model of the human epidermis^103^	Non‐standard models can be used for the same applications as commercial in vitro systems, with the advantage of tunability. Applications which do not have standardized models include fully‐immunocompetent systems, innervated or vascularized systems, and hair follicle research	Technical difficulty (vasculature, innervation, hair follicles, subcutis), not fully validated

* HSE, human skin equivalent.

Beyond clinical application, HSEs are also used for commercial applications (in vitro testing/diagnostics) for testing permeability, sensitization or toxicity studies (Figure 2).^10^ Typically, these HSEs only contain 2 or 3 cell types, keratinocytes and fibroblasts, and sometimes melanocytes.^10^ Challenges with these systems include their differences from skin biology which impacts permeability and barrier functions, difficulty in recapitulating disease conditions or non‐intact skin, and issues with biomaterial choices for the dermis; collagen hydrogels undergo contraction, deterioration, and can have homogeneity and reproducibility issues.^10^, ^14^, ^15^, ^16^ Alternatives to in vitro HSEs include human explant tissue or animal models, however, there have been major efforts to develop relevant in vitro systems that circumvent ethical concerns, biological differences (animal vs human) and donor variation from explant tissue.^17^, ^18^ In vitro systems also provide opportunities to develop controlled experimental conditions or patient‐specific/genetically engineered models.^19^

Human skin equivalents as research tools are diverse in terms of applications, with wound healing as an example of a dominant focus in skin research. As tissue engineering has advanced, the capabilities of in vitro models have progressed with many formats including skin‐on‐chip (or as part of a multi‐organ‐chip), multi‐compartment 2D or 3D devices, and monolayer or full‐thickness models (Table 3). However, most of these in vitro models still focus on only 2 or 3 cell types, generally keratinocytes and fibroblasts, with or without an additional cell type of interest (melanocytes, neurons, etc). While much of this work has been instrumental to the field, most in vitro tissue models do not address the NIC system because they lack the representative components. Ultimately, to discern the effects of cell types or components on the skin system, more complete in vitro tissue models are needed.

## 2 NEURO‐IMMUNO‐CUTANEOUS (NIC) COMPONENTS IN SKIN AND IN VITRO SKIN MODEL RESEARCH

**Figure 3:**
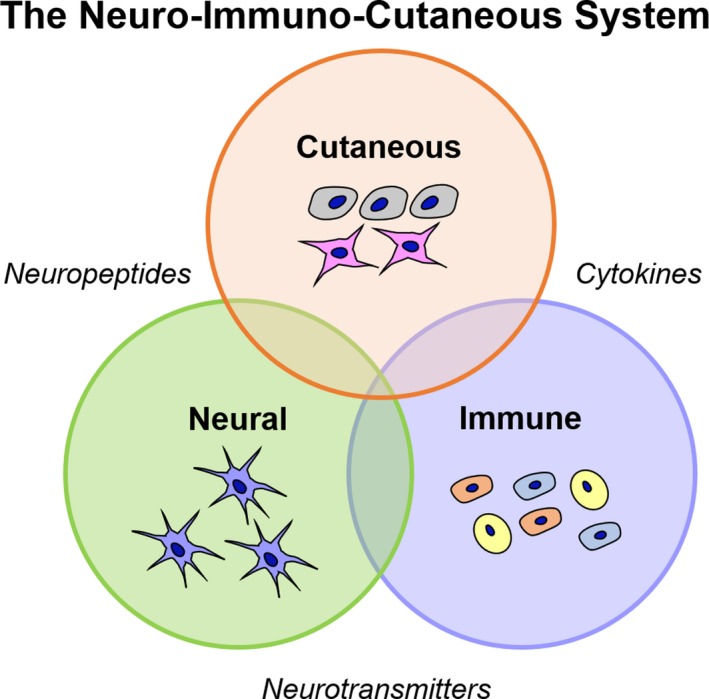
Interconnectedness of the neuro‐immuno‐cutaneous (NIC) system

The NIC system is a relatively new concept for inclusion in in vitro skin model research, although the connection from the brain, skin and host response has been studied with great interest across many fields (psychology, biology, engineering) for many decades.^20^, ^21^ The skin is a key organ to study the connection between the mind, nervous system and the host immune response, as the window to the outside world has tangible links between physical and mental health.^20^ The interconnectedness of the NIC system, and the neuro‐immuno‐cutaneous‐endocrine (NICE) systems, is founded by complex, and constant communication between neuropeptides, cytokines, neurotransmitters, small molecules and less defined processes like psychological stress, to maintain homeostasis in the skin (Figure 3).^21^ Imbalances in stress have been linked to several skin conditions including psoriasis, atopic dermatitis and vitiligo.^20^

Evidence of the interconnectedness and importance of a complex understanding of the skin has been reflected in several recent skin models or HSEs which address some components of the NIC or NICE system (Table 4). However, only one model system has addressed all components simultaneously.^16^, ^22^ However, the human‐induced neural stem cells (hiNSCs) employed by these studies have additional considerations. First, although these are primary human‐derived cells and they express several relevant neuronal markers,^23^ an ideal innervation model would utilize human dorsal root ganglia, which are not readily accessible for in vitro research, thus presenting a challenge.^24^ Therefore, while hiNSCs may be an advantage for the development of all‐human models, they do have limitations as part of an innervation or re‐innervation model.

**Table 4:** Examples of complex HSEs in skin research which address components of the neuro‐immuno‐cutaneous (NIC) or (NICE) systems

Complex HSEs	Models	Descriptions
+ Nerve	Re‐innervated human skin explant^104^ 2D compartmental co‐culture model^32^ 3D HSE with innervation^31^	Human skin explant re‐innervated with rat dorsal root ganglion. Compartmental co‐culture of keratinocytes and porcine dorsal root ganglion HSE (keratinocytes, fibroblast) from collagen matrix innervated with porcine dorsal root ganglion
+ Immune	Microfluidic co‐culture chip^105^ 3D HSE with Langerhans cells^17^	Keratinocyte cell line (HaCaT) and dendritic cell (human leukaemic monocyte lymphoma cell line U937) co‐culture in microfluidic chip design Human Langerhans, keratinocytes and melanocytes in epidermis, with fibroblasts in collagen gel for dermis
+ Adipose	3D skin model with human adipose‐derived stem cells (hASCs)^106^ Two‐layer HSE with pre‐adipocytes and keratinocytes^107^	hASCs seeded into silk sponge as hypodermis, fibroblasts in collagen gel, keratinocytes for epidermis Human pre‐adipocytes seeded onto collagen‐elastin matrix, with keratinocytes seeded on top 4 days later
+Endothelial	3D HSE with endothelial cells^108^ Human in vivo biopsies^85^	Collagen‐based HSE with keratinocytes, fibroblasts, and human umbilical vein endothelial cells form capillary‐like structures Enhancing mechanical support of ageing human skin extracellular matrix via dermal filler has positive effects on fibroblast, endothelial cell and keratinocyte function
+ Combination	3D HSE with neural, adipose, and immune components^16^, ^22^	Silk‐collagen composite gel for dermis containing fibroblasts, epidermis containing keratinocytes, hypodermis component from human lipoaspirate containing adipose and immune cells, with human‐induced neural stem cell coating

Additional complexities to in vitro models can add insights into the NIC system, but it can also contribute complications into full understanding of system contributions (ie whether one cell type alone drives a certain effect, or if the change is systemic). In our recent paper, we identified that through the addition of NIC components (human‐induced neural stem cells, tissue inherent immune, endothelial and adipose cells, in addition to keratinocytes and fibroblasts), that via RNASeq, the groups with and without the NIC components were separate and distinct.^22^

Some skin tissue models which focus on combinations of these components (*eg* nerve and skin cells) can better identify cell‐cell interactions, but limitations to these in vitro approaches remain. Beyond the lack of addressing the full complexity of the NIC/NICE systems in the in vitro design, it would be most desirable to design studies which use only primary, human cells—not cell lines, or animal cells, as the responses may be different than in primary cells. However, with these cell sources, there are certain constraints including donor variability, importance of using low‐passage number cells and the need to optimize media conditions which for some models can be very complex depending on the quantity of distinct cell types.^22^

Therefore, it is important to develop a 3D in vitro HSE, containing only human cells, with the NIC or NICE components to gain a deeper understanding of full cell‐cell interactions or pathways that may have importance in terms of skin diseases with known NIC interactions. For example, numerous skin tissue models containing immune cells have been designed to investigate allergens,^17^ UV‐induced immune events^25^ and sensitization.^26^ These models contributed useful information towards the understanding of skin pathologies including atopic dermatitis, cancers or drug development.

The addition of other NIC/NICE components (approaching full biological relevance and complexity of the skin) allows for enhanced context of atopic dermatitis, without the donor variability or animal ethics concerns that human explant or animal models present.

## 3 PERIPHERAL NERVE ANATOMY IN SKIN

Peripheral nerves consist of 2 types: afferent nerves (directed towards the central nervous system) and motor nerves (towards the peripheral nervous system). There are 3 main types of nociceptive nerve fibres: Aβ, Aδ and C fibres.^27^ Nerve fibres refer to the axon of a nerve, which are responsible for conducting electrical impulses. The thickness of the myelin sheath will differ, which in turn changes the rate of impulse travel. Thicker myelin sheaths relate to faster impulses. Cutaneous sensory nerves are characterized by their cell body size, axon diameters, degree of myelination and conduction velocity.^9^

### 3.1 Aβ nerve fibres

Aβ nerve fibre receptors are located in several areas: Meissner's corpuscles (glabrous skin), Pacinian corpuscles (subcutaneous tissue), Merkel's discs (skin, hair follicles) and Ruffini's corpuscles (skin).^9^ These fibres generally have a low threshold for static and dynamic touch, vibration, and skin stretching, as well as having fast conduction speeds due to heavy myelination.^9^

### 3.2 Aδ nerve fibres

Aδ nerve fibres consist of two subtypes: Type I and Type II.^27^ Type I are high‐threshold mechanical nociceptors, with a high‐heat threshold. Type I fibres respond to mechanical and chemical stimuli, and when injured, their heat threshold lowers, referred to as sensitization. Type II fibres have a high‐mechanical threshold and low‐heat threshold and mainly respond to intense mechanical stimuli. In general, Aδ fibres are thinly myelinated and found in all regions of the skin. Aδ fibres are most associated with localized pain and light touch.^27^

### 3.3 C nerve fibres

C nerve fibres are the smallest and most abundant subtype of nerve, unmyelinated and located in all regions of the skin.^27^ C fibres are free nerve endings, generally referred to as nociceptors that respond to noxious mechanical or hot/cold stimuli^9^ Impulses travel slowly in C fibres and are associated with poorly localized, slower pain.^27^

### 3.4 Neurons interaction with keratinocytes, dermal fibroblasts

The epidermis is populated with fine, unmyelinated nerve endings, and free, branched nerve endings in the dermis.^28^ Neurites interact with other skin cells in several ways. Peripheral nerves originate from dorsal root ganglia of the spinal cord and send neurites through the dermis into different locations in the skin including the dermo‐epidermal junction and are highly sensitive to other skin cells in this microenvironment and can adjust neurite growth accordingly.^6^, ^24^, ^29^, ^30^ Neurites are known to form close membrane associations with some dermal cells (fibroblasts), but this is not the case with epidermal cells, suggesting there are preferential and cell‐dependent interactions of neurons with other skin cells.^29^ Neurons have induced proliferation in keratinocytes, which could be reversed by inhibiting calcitonin gene‐related peptide (CGRP).^31^ Fibroblasts and keratinocytes secrete distinct levels of NGF and cerebral dopamine neurotrophic factor (CDNF), which in an in vitro study demonstrated changes in neurite morphology and axonal neuropeptides.^32^ Atopic keratinocytes can enhance neurite outgrowth and the resultant CGRP‐positive nerve fibres through elevation of NGF.^32^ Separate studies have demonstrated that by adding nerves to an HSE, there is a thickening of epidermis from keratinocyte proliferation due to cell‐cell communication between nerves and keratinocytes.^31^

## 4 PERIPHERAL NERVE INTERACTION IN SKIN

Peripheral nerves may also communicate with other cell types in the skin through alternative signalling pathways such as cytokine signalling, neurotrophins or neuropeptides. Neuropeptides are expressed widely by many cell types of the skin including keratinocytes, fibroblasts, Langerhans cells, endothelial cells and immunocytes.^20^

### 4.1 Acetylcholine (Ach)

Acetylcholine (Ach) is synthesized in nerve terminals from acetyl coenzyme A and choline, it is an excitatory neurotransmitter.^33^, ^34^, ^35^ Acetylcholine acts as an immune cytokine, which inhibits macrophages through cholinergic (receptors which respond to acetylcholine) anti‐inflammatory pathways.^34^ Several nicotinic Ach receptor (nAChR) agonists have been developed to treat subcutaneous inflammation due to the relationship of various immune cells (such as monocytes and macrophages) with nAChRs. Macrophage nAChRs can modulate functional activity of cholinergic anti‐inflammatory pathways which regulate innate immune function and inflammation.^34^ Keratinocytes have been shown to produce non‐neuronal acetylcholine.^36^ Additional evidence, such as sensory receptors located on keratinocytes, suggests that they are also sensory cells.^6^, ^37^

### 4.2 Neurotrophins in the skin

Neurotrophins in the skin have roles in both early and late stages of the inflammatory response. Cutaneous neurotrophins are expressed by sensory, sympathetic neurons and non‐neuronal cells which relate to functions in nerve growth and development, apoptosis, epidermal homeostasis, inflammation, wound healing and hair growth.^8^ Neurotrophin receptors can be located on sensory nerves, keratinocytes, melanocytes, fibroblasts, mast cells, immune cells and hair follicles.^8^ Neurotrophins can be induced by cytokines and are produced by many cells both sensory neuronal and skin cells (keratinocytes, fibroblasts and immune cells). Neurotrophins 3, 4 and 5 are all essential for growth, proliferation and maintenance of nerves. Neurotrophin 3 is responsible for the development of cutaneous nerves and promotes the survival of cutaneous sensory nerves.^6^, ^8^ Common neurotrophins include nerve growth factor (NGF), brain‐derived neurotrophic factor (BDNF) and neurotrophins 3, 4, 5.^38^ In general, neurotrophins are key molecules in neuro‐immuno‐endocrine signalling.^4^

### 4.3 Neuropeptides

Neuropeptides are secreted by cutaneous nerves and can interact with many cutaneous cell types including keratinocytes, Langerhans and endothelial cells.^39^ Sensory neurons secrete at least 17‐20 different neuropeptides, including substance P (SP), neuropeptide Y (NPY) and nerve growth factor (NGF).^39^, ^40^

#### 4.3.1 Substance P

Substance P is secreted by sensory C fibres,^41^ dorsal root ganglion,^38^ can bind to keratinocytes, mast cells,^28^ or induce interleukin (and other cytokine) release.^39^ There are 3 main peripheral actions of SP: vasodilation or vascular permeability, local inflammation or immune system effects, and increased cellular proliferation (keratinocytes, fibroblasts, endothelial cells and immune cells).^41^ SP has also been implicated in psoriasis.^4^

#### 4.3.2 Neuropeptide Y (NPY)

Neuropeptide Y is a neuronal signalling molecule^42^ which is a part of the NIC system that can act locally (ie inflammation) or act on entire systems (via endocrine or neuro‐endocrine pathways).^6^ NPY has several functions, including activating mast cells, induction of phagocytosis, stimulating antibodies and cytokines, and inducing vascular permeability.^4^

#### 4.3.3 Nerve growth factor (NGF)

Nerve growth factor is secreted by several cutaneous cells including keratinocytes, fibroblasts, nerves and adipocytes.^8^, ^43^, ^44^, ^45^, ^46^, ^47^, ^48^ NGF is known to mediate cutaneous re‐innervation^41^ and is released in high concentrations during inflammation. NGF is responsible for the maintenance, proliferation and growth of nerve cells. During cutaneous inflammation, there is NGF‐dependent production of SP, CGRP, sodium channels, and other neurotransmitters and neuropeptides or molecules related to nociception.^38^ NGF also promotes the survival of several immune cells in the cutaneous system including eosinophils, monocytes, neutrophils, T cells, macrophages and basophils.^38^ There is a clear link between NGF and the immune system for the response and survival of several immune cells as well as modulating cell behaviour. NGF is highly upregulated in cutaneous nerves following an inflammation event; NGF has also been linked to psoriasis.^8^

## 5 DYSREGULATION OF NEURO‐IMMUNO‐ENDOCRINE SYSTEMS IN SKIN PATHOLOGIES

The immune system of the cutaneous system is extremely important as the first line of defence of the body against the environment and is composed of numerous cell types that are distributed throughout the skin in key locations for their function.^49^ There has been significant advancement into the understanding of the complexity of the immune cells and dendritic cells which populate the skin.^50^, ^51^, ^52^ While there is much information known, there is still exciting work to be done in discovery of the immune and neural systems of the skin to fully integrate of our understanding of the NIC/NICE systems with various skin pathologies (Table 5). Dysregulation of components of the NIC/NICE systems has been implicated in numerous skin pathologies.

**Table 5:** Skin pathologies in humans with complex connections to NIC/NICE system or components

Skin pathology	Brief definition	NIC/NICE system linkage	Description
Psoriasis vulgaris	Accumulation of inflammatory cells in the epidermis and hyperproliferation of keratinocytes resulting in thickened epidermis often in pruritic scales or patches	Neural Immune Endocrine	Psoriatic plaques may contain high nerve density with alteration in neuropeptide (SP, CGRP, NGF) activity 6, 8, 28, 109, 110 Immune‐related inflammatory disease, driven by activated T cells^68^, ^111^; Pro‐inflammatory proteins and exposure to chronic stressors may dysregulate stress‐immune response^56^, ^109^ Hormone‐mediation: glucocorticoids, epinephrine, thyroid hormones, insulin^112^ [Roman 2016]
Atopic dermatitis	Characterized by chronic inflammation or itch	Neural Immune Endocrine	Epithelial cells communicate with neurons to induce inflammation (itch) via cytokine thymic stromal lymphopoietin (TSLP); activation may be direct to neurons or indirect via immune cells^113^, ^114^, ^115^ Impaired cutaneous barrier function combines with higher sensitivity to environmental stressors with effects on the immune response,^110^ chronic inflammation disorder Highly sensitive to glucocorticoids, hyper‐reactive to stress‐induced cortisol^110^
Vitiligo	Depigmentation of the skin in patches that is often progressive	Neural Immune Endocrine	May be related to neuronal interaction with melanocytes, or dysfunction of neurons or neuropeptides^116^, ^117^ Autoimmune component mediated by cells, antibodies, or cytokines 117, 118, 119, 120 Hormonal or stressor‐related 118, 121

Based on research into the effect of neuromediators, it has been suggested that they do not simply play a pro‐inflammatory role in the skin but also can and do participate in the entire inflammatory response process.^40^ Cutaneous innervation or immune components are targets for many treatments of disease; as a result, promising results have been gained from developments in therapeutics from a neuro‐immuno‐endocrine approach: neuropeptides like NGF,^53^, ^54^ hormonal (vitamin D),^55^ anti‐cytokine 56 and capsaicin to target sensory neurons of the skin.^57^, ^58^, ^59^ Continuing emphasis on the NIC/NICE systems for multi‐pronged treatments of these pathologies could be an important consideration for drug development and reveal further intricacies of the skin.

## 6 CONCLUSIONS AND OUTLOOK

The ability to design HSEs that include all components of NIC/NICE systems would advancement the field by enhancing the understanding and treatment of numerous skin pathologies or conditions. Currently available clinical, commercial and research models of the skin in general are limited to a few cell types (keratinocytes and/or fibroblasts) or layers (epidermis and/or dermis) and may not be relevant to the full complexity of the human skin. Further, the biomaterial choice for dermis materials does not fully reflect the mechanical environment, extracellular matrix requirements and functional biological similarity to be reliable HSEs.^10^, ^14^, ^15^, ^16^ As the skin is composed of a multitude of ECM components including collagen types I, III and IV, elastin, fibronectin and proteoglycans, placement of these biomaterials to recapitulate skin layers is also important (ie collagen type I as the main dermal component, collagen type IV as basement membrane material substrate), an ideal skin biomaterial would be correspondingly complex and spatially distributed to encourage the proper differentiation of cells.^10^, ^24^, ^60^

In summary, the most important considerations for the design of an optimal skin biomaterial would be the following: mechanically robust and/or flexible to allow for skin movement, biodegradable at controlled rate to optimize integration with cells or the local tissue (for implants), bio‐inertness of the scaffold, conductivity to aid neural integration and/or reintegration, and additional factors to promote healing to enhance neural regrowth and/or wound healing.^24^, ^61^

A crucial avenue of skin research, and of relevance to the innervation field, is wound healing. Neuromediators are involved in all stages of the wound‐healing process, and it is crucial to consider the potential loss of sensation, innervation and re‐innervation following trauma such as a burn, a common consequence following injury.^30^, ^61^, ^62^ Future work could address the lack of medical treatment options for re‐innervation of skin following burn injuries.^61^ One way to expand research in this area would be to investigate ‘bio‐active’ materials for skin tissue replacements or in vitro skin tissue models which enhance healing, regrowth of neural cells, and also include or reintegrate vasculature.^24^, ^61^ Bioprinting is another approach to create bio‐active skin with innervation and/or vascularization architectures within biomaterials, however, despite advances in skin bioprinting, such materials have yet to be developed for skin.^61^, ^63^, ^64^

Recent research demonstrated that cutaneous cell types have complex communication that reaches across systems, *that is* keratinocytes (cutaneous) interact with neurons (neuronal system) and vice versa.^1^, ^47^, ^65^, ^66^ Several skin pathologies have known interactions between skin cells like keratinocytes, with neuronal and/or immune cells including psoriasis, atopic dermatitis and vitiligo.^67^, ^68^

The understanding of the impact of the NIC/NICE system on skin pathology is just at the beginning. Through developments in in vitro HSE design, by inclusion of NIC/NICE components, it would be possible to gain insights into human pathologies in a manner that avoids animal testing and is more translatable to human biology. Complete, complex in vitro HSEs could become fully viable alternatives to animal testing, increase accuracy of in vitro testing models, and serve as sensory and immunocompetent disease models.^5^, ^69^

## Data Availability

Data sharing is not applicable to this article as no new data were created or analysed in this study.
